# Temporal Trends in Satellite-Derived Erythemal UVB and Implications for Ambient Sun Exposure Assessment

**DOI:** 10.3390/ijerph14020176

**Published:** 2017-02-10

**Authors:** Marvin Langston, Leslie Dennis, Charles Lynch, Denise Roe, Heidi Brown

**Affiliations:** 1 Department of Epidemiology and Biostatistics, Mel and Enid Zuckerman College of Public Health, University of Arizona, Tucson, AZ 85724, USA; ldennis@email.arizona.edu (L.D.); droe@email.arizona.edu (D.R.); heidibrown@email.arizona.edu (H.B.); 2 Division of Public Health Sciences, Department of Surgery, Washington University in Saint Louis School of Medicine, St. Louis, MO 63110, USA; 3 Department of Epidemiology, College of Public Health, University of Iowa, Iowa City, IA 52246, USA; charles-lynch@uiowa.edu

**Keywords:** ultraviolet radiation, exposure assessment, melanoma, sun exposure

## Abstract

Ultraviolet radiation (UVR) has been associated with various health outcomes, including skin cancers, vitamin D insufficiency, and multiple sclerosis. Measurement of UVR has been difficult, traditionally relying on subject recall. We investigated trends in satellite-derived UVB from 1978 to 2014 within the continental United States (US) to inform UVR exposure assessment and determine the potential magnitude of misclassification bias created by ignoring these trends. Monthly UVB data remotely sensed from various NASA satellites were used to investigate changes over time in the United States using linear regression with a harmonic function. Linear regression models for local geographic areas were used to make inferences across the entire study area using a global field significance test. Temporal trends were investigated across all years and separately for each satellite type due to documented differences in UVB estimation. UVB increased from 1978 to 2014 in 48% of local tests. The largest UVB increase was found in Western Nevada (0.145 kJ/m^2^ per five-year increment), a total 30-year increase of 0.87 kJ/m^2^. This largest change only represented 17% of total ambient exposure for an average January and 2% of an average July in Western Nevada. The observed trends represent cumulative UVB changes of less than a month, which are not relevant when attempting to estimate human exposure. The observation of small trends should be interpreted with caution due to measurement of satellite parameter inputs (ozone and climatological factors) that may impact derived satellite UVR nearly 20% compared to ground level sources. If the observed trends hold, satellite-derived UVB data may reasonably estimate ambient UVB exposures even for outcomes with long latency phases that predate the satellite record.

## 1. Introduction

Ultraviolet radiation (UVR) exposure and health outcomes including skin cancers, multiple sclerosis, and vitamin D insufficiency have been associated in recent epidemiological research [1]. Although the harms and benefits of UVR have been previously described, measurement to understand the harmful dose has proven difficult [2,3]. Assessing and quantifying human solar UVR exposure involves measuring complex environmental and human host factors and their interaction [4]. Contributing environmental factors include atmospheric ozone, absorbing aerosols in the atmosphere, and cloud cover on a particular day [5]. Host factors include human skin sensitivity including skin color, gender, and age, along with host risk factors of relative time spent outdoors in the sunshine and number of sunburns (possibly representing damage reflected by UVR and skin sensitivity) [6]. UVR from the sun is comprised of UVA (λ = 320–400 nm), UVB (λ = 280–320 nm), and UVC (λ = 200–280 nm). UVC is not relevant since it is typically absorbed by ozone in the upper atmosphere. UVB can be absorbed by human skin causing sunburns, while UVA may be absorbed by deeper subcutaneous tissues. UVB has the potential for more cutaneous skin damage [6] and is weighted more heavily than UVA in the action spectrum that determines the UVR index [7].

In order to determine risk for UVR-related outcomes, most investigators rely on subject recall of sun exposure, sunburns, and other factors, which are imprecise [8,9]. Due to the long latency of many UVR-associated outcomes, accurate exposure assessment requires detailed accounting of UVR exposures going back decades [9]. Approaches solely using sun sensitivity factors combined with self-report ignore the additional complexity of environmental and atmospheric components that drive the variation in UVR.

Another approach to UVR exposure assessment has been through the use of latitude as a proxy for ambient UVR or potential sun exposure. Unlike subject recall, latitude provides an objective estimate. Previous studies have found associations of latitude with multiple sclerosis [10,11,12], melanoma [13,14], and non-melanoma skin cancers [15]. However, these studies do not capture intra-latitude variability between places, such as South Carolina and Arizona, two states within the same latitude, but with very different sun exposures [16]. Furthermore, latitude use does not estimate actual ambient UVR exposure.

The National Solar Radiation Database (NSRAD) produced by the National Renewable Energy Lab has been measuring ground level UVR since 1960. UVR is measured at meteorological stations across the country with some satellite modeling (1998–present). However, only 239 of 1454 stations have recorded UVR before 1990 and many of these stations do not have continuous or reliable estimates tracing back to 1960 [17,18]. Furthermore, while some populations in the United States (US) are close to their nearest station, others are more distant. In order to provide spatially continuous gridded data, interpolations have been made based on these point locations [17]. Interpolated estimates of ground level UVR from stations have been shown to be less accurate over long distances than satellite-derived estimates using an estimated ground truth [19]. With interpolated data, seemingly small but clinically relevant variations may be lost depending on the density of the stations and interpolation methods used, especially in areas where data coverage is less pronounced, such as some Western US states [20].

The National Aeronautics and Space Administration (NASA) has been providing remotely sensed estimates of UVR onboard various satellites using Total Ozone Mapping Spectrometers (TOMS) since 1978 as an alternative to ground level, discontinuous measures. This allows for spatially-continuous gridded UVR data without the disadvantage of large scale interpolation. Unlike ground level instruments, the satellite data provide continuous standardized estimates across the globe which allows for study of UVR exposures outside of the US. Satellite data have been used in the study of various health outcomes including multiple sclerosis [16], melanoma [21,22,23] and other cancers [24,25,26,27,28,29,30], asthma and hayfever [31], diabetes [32], and mortality [33,34]. Although satellite data represent an opportunity for standardized UVR estimation across vast distances many criticisms for use of this source exist, such as data validation and seasonal inconsistencies [35]. Another criticism of satellite-derived UVR data is the relatively short record (1978–present), which makes exposure assessment difficult for outcomes with long latency periods. This latency could include childhood and young adult exposures that have been shown to be important in the development of diseases in older individuals [9,36,37,38,39]. No estimation of satellite-derived ambient UVR exposure in individuals with UVR exposure history prior to the satellite record is available. Previous studies have used post 1978 UVR data as proxies for pre-1978 exposures. Use of solely post 1978 exposure data could potentially lead to misclassification of the exposure if UVR is changing appreciably over the life-course.

The current study investigates trends in UVB using available satellite data (1978–2014) to generate inferences for UVB over time in the US, uses modeled UVB trends to inform prior assumptions of pre-1978 UVB, and makes recommendations for UVB human exposure assessment in future studies.

## 2. Materials and Methods

Solar UVB data were obtained from NASA’s TOMS [40] and the Ozone Monitoring Instrument (OMI) [41]. The TOMS system has been collecting ground-level UVB data aboard Nimbus 7 from 1978 to 1993 and aboard the Earth Probe satellite from 1996 to 2004. Since October 2004, the OMI, which is onboard the NASA EOS Aura satellite, has continued the recording of TOMS. Both systems provide continuous UVB data across the entire globe on 1° latitude × 1.25° longitude grid resolution for TOMS and 1° × 1° degree resolution for OMI. In the mid-latitudes of the continental US, one degree is about 69 m north to south and depending on latitude, 47–63 m east to west.

UVB estimates were daily noontime ground-level irradiances that took into account cloud conditions, ozone column, length of day, solar zenith angle, surface albedo (snow or forest cover), and atmospheric aerosols. Additionally, the UVB data used for this analysis were erythemically-weighted, which means they were weighted by the reference erythemal action spectrum, a measure of potential for UVB biological damage to the skin erythema (skin redness) of Caucasians [7]. OMI uses a similar algorithm to estimate UVB as TOMS, but has additional corrections for surface albedo to improve the underestimation of UVB at high latitudes due to the interpretation of cloud cover vs. snow cover [42,43]. The erythemal UVB calculated by TOMS/OMI is consistent with the standard set by the Commission Internationale de l’ Éclairage [7,44]. TOMS data algorithm version 8 was used to estimate erythemal UVB [45]. This 30-year record of UVB data provided by TOMS and OMI was used for our analyses. TOMS/OMI have been previously validated and found to mostly overestimate UVB compared to ground level sensors [46,47,48,49,50,51,52]. Any analysis using TOMS/OMI satellite data in combination must be carefully interpreted due to inter-satellite variability and widespread validation differences between satellite and ground based sensors.

### Statistical Analysis

For each 1° × 1° grid square across the continental US, daily UVB estimates were averaged by month for OMI by summing each daily estimate and dividing by the total number of non-missing daily UVB observations for the month. The continental US was used as the unit of observation and will be referred throughout as the US. OMI monthly data were re-gridded using bi-cubic interpolation to match the lower resolution TOMS sensors. A total 392 months of UVB data from November 1978 to December 2014 were used for analysis (174 from 1978 to 1993, 96 from 1996 to 2004, and 122 from 2004 to 2014). Due to data availability and reliability issues, UVB data from May 1993 to July 1996 were not included in this analysis which includes data from TOMS Meteor-3 (August 1991–December 1994) satellite. Average annual UVB exposure by grid was calculated as the sum of the twelve monthly averages for the time period 1978–2014.

Using general linear modeling, we examined the mean difference in UVB over time for each grid square. This comparison allows us to assess the stability of UVB estimates over the full study period (crude analysis). We used the following linear regression model:

UVB_Gridpoint(i,j)_ = β_0Gridpoint(i,j)_ + β_1Gridpoint(i,j)_Year + β_2Gridpoint(i,j)_sin(2πMonth/12) + β_3Gridpoint(i,j)_cos(2πMonth/12) (1)

where β_1_ represents the estimated change in UVB by year, and β_2_, β_3_ account for the seasonal cyclical trends in UVR by month. These last two terms in the model represent the temporal autocorrelation by month using a harmonic function that runs a full cycle in one calendar year. The harmonic function was included to account for temporal autocorrelation [53,54,55], which is expected in the data due to cyclical trends in UVB over the course of a year. UVB peaks in the summer months and reaches its minimum value in the winter months within the US. This autocorrelation violates the independence assumption of linear regression and can be seen by large correlation coefficients between regression residuals and 1- or 12-month time lags.

While OMI was introduced to continue the longitudinal satellite estimation of UVR initiated by TOMS, these systems represent distinct time-series that make longitudinal analyses difficult to conduct and interpret. This is due to reported differences between satellite sensors in initial pixel size [56], surface albedo climatology [42], aerosol [35] and cloudiness correction [56,57], and estimates of total ozone [35]. Therefore, in addition to the crude analysis across the full study period, UVB data were grouped and compared based on the three satellite sensor combinations (A) TOMS aboard Nimbus-7 satellite (1978–1993); (B) TOMS aboard the Earth Probe satellite (1996–2004); and (C) Ozone Monitoring Instrument (2004–2014). Trends in erythemal UVB were examined separately within each grouping for the stratified analysis using Equation (1).

Due to potential confounding of the relationship between the time period and detected erythemal UVB, adjusted models were explored with the satellite type as a proxy for time period. For these models the linear regression model (Equation (1)) additionally includes terms for type of satellite sensor.

We used linear regression to examine the magnitude of UVB change over time independently for each grid square, which we call the local tests. To account for multiple testing and spatial autocorrelation of local significance tests, we used a field significance test to determine if UVB changed statistically over the study area based on the results of the local tests. This field significance test, which helped make less biased inferences from the local linear regression, we call the global test [58]. For the global test, grid points with locally significant differences were counted and compared to a null distribution generated using Monte Carlo techniques with random permutation (*n* = 1000) with replacement of all available years of data. Two tailed *p*-values < 0.05 were considered significant for both local and global tests. Analyses were conducted in MATLAB R2013b (The MathWorks Inc., Natick, MA, USA). Data visualization and map creation were conducted in ArcGIS 10.1 (ESRI, Redlands, CA, USA).

## 3. Results

**Figure 1:**
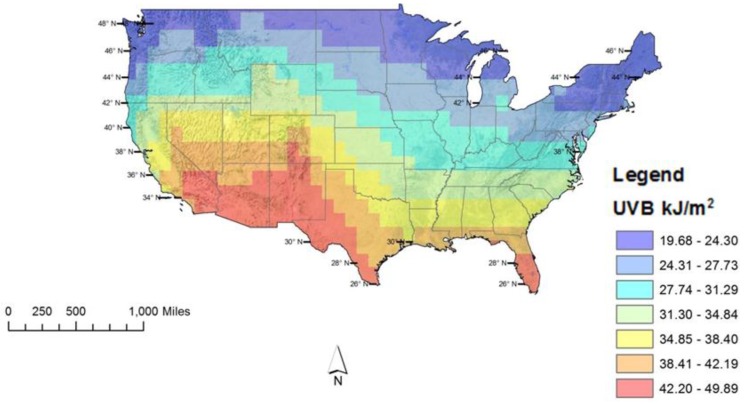
Yearly Potential UVB Dose. Erythemal UVB observations by location were averaged by month from 1978 to 2014. These averages were subsequently combined to represent a yearly potential dose.

From 1978 to 2014, the average cumulative erythemal UVB ranged from 19.68 kJ/m^2^ in the northeastern and northern US to 49.89 kJ/m^2^ in the southwestern region and Southern Florida (Figure 1). As expected, these patterns in yearly UVB followed latitude lines closely. UVB was also greater in areas of higher elevation. Greater variability was observed in areas of high relief where the difference between high points and low points in the terrain were the most pronounced.

**Figure 2:**
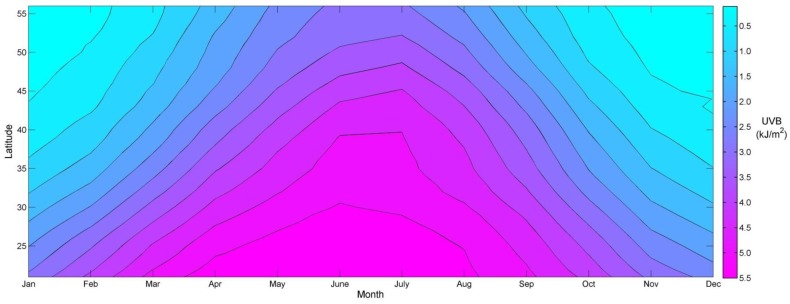
Average monthly erythemal UVB (kJ/m^2^) in the mid-latitudes, 1978–2014. Contours represent a 0.5 kJ/m^2^ change in UVB from one contour to the next.

**Figure 3:**
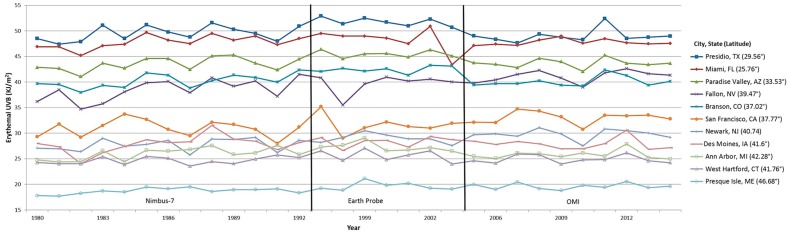
Annual erythemal UVB for selected cities. Displays the annual erythemal UVB for selected cities based on (1) high (Fallon, NV and Branson, CO) and low (Ann Arbor, MI and San Francisco, CA) beta values from the adjusted model representing the most extreme UVB changes seen over the study period; and (2) maximum (Presidio, TX), moderate (Des Moines, IA) and minimum (Presque Isle, ME) average cumulative erythemal UVB. The remaining cities were selected randomly from all available large US cities (>50,000 residents in 2007) based on the US Census. Years without 12 months of available UVB data are not included here (1978–1979, 1993–1996, 2004).

The mean erythemal UVB followed a cyclical trend by month that peaked in the summer months and reached a minimum in the winter as expected (Figure 2). Within the US, monthly UVB ranged from 0.112 kJ/m^2^ in January in Maine to 5.902 kJ/m^2^ in July in Florida. We selected several US cities in order to visually examine the average cumulative UVB trends more closely (Figure 3). Based on these cities, average cumulative UVB remained fairly constant over the study period with changes ranging from 5.3 kJ/m^2^ to 7.9 kJ/m^2^.

**Figure 4:**
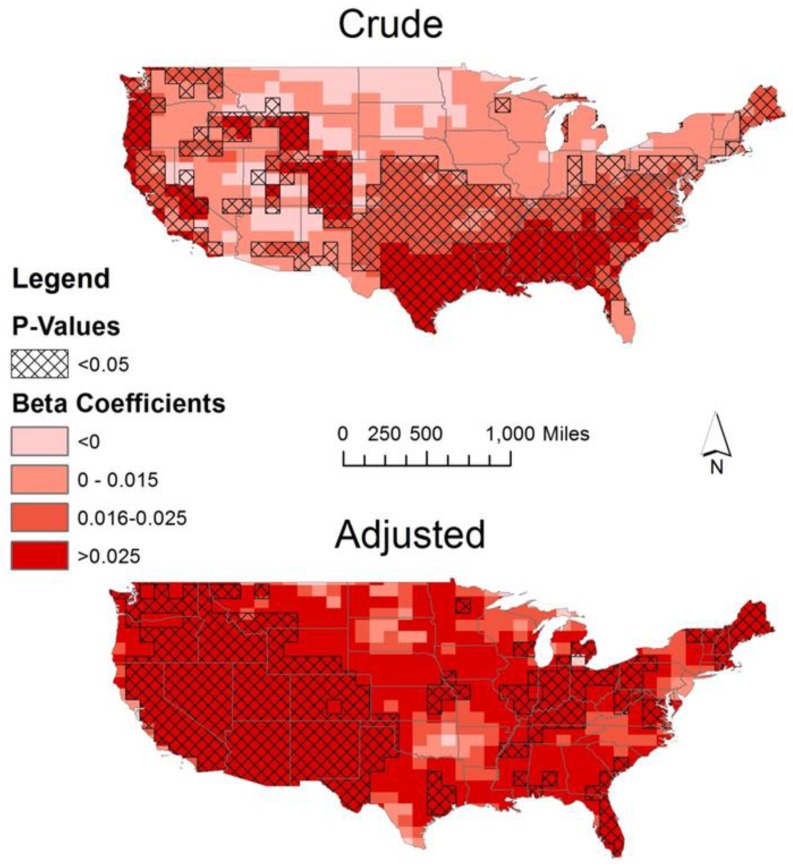
Trends in erythemal UVB. Local linear regression of erythemal UVB by location from 1978 to 2014 at α = 0.05 for the crude model and adjusted for satellite (Nimbus-7 satellite 1978–1993, Earth Probe satellite 1996–2004, and the Ozone Monitoring Instrument 2004–2014). The beta coefficients shown represent the estimated effect of a 5-year increase on the mean erythemal UVB by location. Beta coefficient symbology based on quartiles of the crude analysis with adjustment in quartile 1 for positive value cutoff.

**Figure 5:**
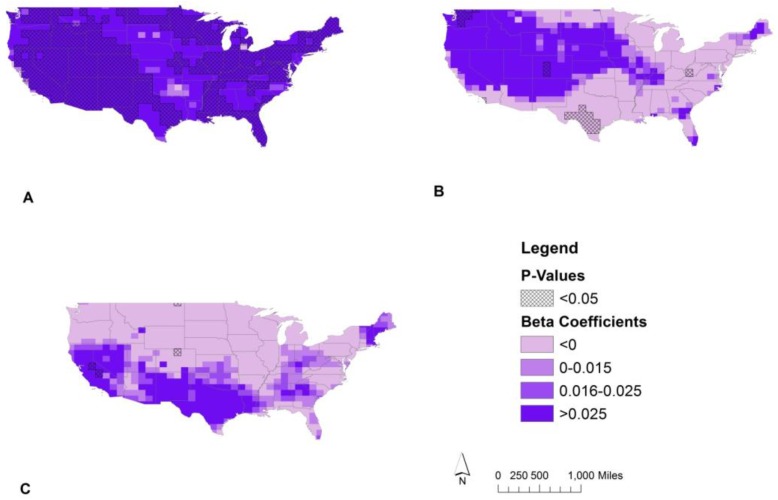
Trends in erythemal UVB stratified by satellite type. Local linear regression of erythemal UVB by location from 1978 to 2014 at α = 0.05 stratified by satellite. The beta coefficients shown represent the estimated effect of a 5-year increase on the mean erythemal UVB for (**A**) Nimbus-7 satellite 1978–1993; (**B**) Earth Probe satellite 1996–2004; and (**C**) the Ozone Monitoring Instrument 2004–2014. Beta value symbology based on quartiles of the crude analysis with adjustment in quartile 1 for positive value cutoff.

Temporal trends in UVB were symbolized by combining the trend estimates into equal quartiles for the maps created (Figure 4 and Figure 5). The cutoff value for the lowest quartile of UVB trend estimates was set a priori in order to appropriately distinguish decreasing UVB over time. The initial temporal analysis over all years from 1978 to 2014 identified some areas of UVB change. These changes occurred mainly in the southeast, Texas, and along much of the east and west coast regions (Figure 4). UVB increased in 69% of local tests, decreased in <1%, and did not change in 30%. The global test indicated that a statistically significant change in UVB occurred during this time period, but the magnitudes of change indicated by the local linear regression models were small (−0.215 to 0.163 kJ/m^2^ per five-year increment). In magnitude this amounts to less than the January (one month) average ambient UVB exposure in all US locations (Figure 2).

Adjusting for satellite type, the changes in erythemal UVB were seen in the much of the western half of the country, the Appalachian Mountains, along Lake Erie, New England, and the Atlantic coastline of the southeastern states (Figure 4). A five-year increase in this adjusted model translated to a 0.001 to 0.145 kJ/m^2^ increase in erythemal UVB. UVB increased over time in 48% of local tests and did not change in 52%. There were no significant decreases in UVB. As with the crude model, the global test indicated a significant change in UVB over time, but the magnitude of this change was small based on the results of the local linear regression models.

The model stratified by the satellite type (Figure 5) revealed a more nuanced spatial pattern for UVB change over time. From 1978 to 1993, TOMS aboard Nimbus-7 showed small increases in UVB (0.007 to 0.038 kJ/m^2^ per five-year increment) based on local linear regression tests, most notably in the western half of the US, along the southeastern coastal region, the midwest and northeast (Figure 5A). Results of the global test indicated a significant change in UVB for 1978–1993 TOMS data, but the magnitude of change was small as previously stated. From 1996 to 2004 for TOMS onboard the Earth Probe satellite (Figure 5B) decreases in UVB were seen in parts of south central West Virginia including the Allegheny Mountains (−0.100 to −0.030 kJ/m^2^ per five-year increment), Southwest Texas (−0.265 to −0.075 kJ/m^2^ per five-year increment), and the Southern California coast near San Diego (−0.210 to −0.165 kJ/m^2^ per five-year increment). The largest increases in UVB for 1996–2004 were along the southern Rocky Mountains in Colorado (0.095 to 0.150 kJ/m^2^ per five-year increment) and near the Cascade Mountains in Washington (0.115 to 0.195 kJ/m^2^ per five-year increment). From 2004 to 2014, the OMI model shows small decreases in UVB in Northeastern Montana (−0.095 to −0.085 kJ/m^2^ per five-year increment) and north central Colorado (−0.060 to −0.010 kJ/m^2^ per five-year increment) (Figure 5C). The largest increases in UVB for 2004–2014 were along parts of the Sierra Nevadas (0.065–0.090 kJ/m^2^ per five-year increment). Although some local tests were significant, results of the global tests for both 1996–2004 and 2004–2014 time periods showed no significant changes in erythemal UVB across the study area. Therefore, any interpretations of UVB change from these two stratified models (Figure 5B,C) should be made only at the local level.

## 4. Discussion

UVB changed in various locations across the study area from 1978 to 2014. The largest local change was observed in Fallen, Nevada (0.145 kJ/m^2^ per five-year increment) represented 17% of total ambient exposure for an average January and 2% of an average July in this area. In Maine and Western Texas, the areas of lowest and highest cumulative UVB, respectively, this is equivalent to 49% of one month of January ambient exposure in Maine and 2% of one month of July ambient exposure in Western Texas. The small changes in erythemal UVB over the observation period were well within the range of average monthly UVB variation in the US. Such misclassification would then be within the range of an extra month of exposure (or one less month) reported over a lifetime. Thus, the observed trends have only a small impact on long-term human UVB exposure assessments.

The largest erythemal UVB increases from 1978 to 2014 in the model accounting for satellite type were found in parts of Western Nevada (0.145 kJ/m^2^ per five-year increment). Over a potential 30-year period, UVB would increase by 0.87 kJ/m^2^. This is equivalent to one adding a January month of exposure for Iowa (Figure 2 at 41.6° latitude for Iowa). Current approaches to UVB exposure assessment that consider lifetime UVB exposures would render this exposure misclassification as negligible when considering the dose required for some outcomes, 0.5 kJ/m^2^/day increase of average annual UVR to decrease multiple sclerosis prevalence from 28 to 25 per 100,000, or the magnitude of changes seen in context of monthly US fluctuations in UVB [59].

Recent case-control studies explored the association between lifetime ambient erythemal UVB irradiance for melanoma [21,22] and esophageal cancer [30]. Cust et al. [21] classified exposure groups based on quartiles (Q) of lifetime ambient erythemal UVB exposure (Q1 < 35,462; Q2 = 35,462–42,004; Q3 = 42,005–47,784; Q4 > 47,784 kJ/m^2^) in cases and controls. Similarly, Tran et al. [30] classified groups by tertiles (T) of lifetime ambient erythemal UVB (T1 < 66,678; T2 = 66,981–83,140; T3 > 83,176 kJ/m^2^). For the Nevada increase indicated above, an 80-year-old who lived his/her whole life in Western Nevada, the maximum increase seen in the 1978–2014 NASA data would be equivalent to a lifetime ambient erythemal UVB increase of 2.32 kJ/m^2^. Based on categories of exposure in these studies, ignoring the maximum increase in erythemal UVB (and smaller increases) that we found would not result in appreciable misclassification of the exposure. Therefore, our observed changes in UVB have only a small impact on long-term human UVB exposure assessments and may not be relevant for future studies using these data.

Our observation of small increases and decreases in erythemal UVB from 1978 to 2014 could be due to the interplay of various factors including ozone depletion, ozone recovery, and climate change (changes in cloud cover, temperature, and surface albedo). Ozone in the stratosphere reduces the amount of erythemal UVB that reaches the surface of the Earth. Some estimates have described a 1000-fold reduction in erythemal UVB by ozone [60]. In the late 1970s depletion of UVB reducing ozone became more important as the incidence of skin cancers began to rise (more than 50% for melanoma from 1974 to 1981) leading to more research in this area to assess causality [61]. The reductions in ozone over large geographic areas became known as ozone holes. The Montreal Protocol, which curbed production of a wide range of ozone depleting substances, began implementation in 1987. Assuming UVR causality, some have predicted that the incidence of skin cancer would have been 14% greater by the year 2030 without the Montreal Protocol [62]. Further estimates have suggested that over the next 30 years increases in erythemal UV radiation will be negligible for the continental US with return to pre-1980s levels in the next ten years [63]. These will be driven by the recovery of atmospheric ozone. The small changes in erythemal UVB demonstrated in this current study from 1978 to 2014 provide additional evidence for a long-term stable trend.

The changes we identified could also derive from errors in satellite estimation. Our stratified models showed small local changes in UVB mostly in geographic areas near mountains and coastal regions. These changes could be due to documented difficulties in satellite erythemal UVB estimation from previous validation studies [48,64,65,66,67,68]. When comparing UVR estimates for satellite to ground level sensors, satellite estimates are more often higher [46,47,48,49,50,51,52]. Daily corrections for cloudiness or total column ozone may not match temporally with UV irradiance calculated at local solar noon [50]. Scattering of clouds and the 3D effects of broken clouds are not appropriately accounted for in the satellite UVR estimates [69]. Diurnal variation in cloudiness is not accounted for causing uncertainty in daily UVR doses of nearly 20% [70,71]. In some instances, satellite sensors are unable to discern accurately between snow and clouds in calculations of surface albedo and cloud cover leading to differences based on seasonality as well [72]. Mountainous regions with high snow cover and largely variable cloud cover are problematic for satellite-derived erythemal UVB estimates [52]. Three-dimensional radiative transfer models may be used to accurately model erythemal UVB near mountains [73]. Absorbing aerosols are not accounted for in satellite UVR estimation, which may disproportionately affect urban areas [64,74,75]. Considering surface albedo, altitude, and aerosols together we can begin to understand the magnitude of their joint effect on satellite UVR estimation. In low altitude, snow-free regions with moderate absorbing aerosols, in general satellites may overestimate UVR by nearly 20% compared to ground level sources [50]. This overestimation could increase to nearly 50% in regions with high absorbing aerosols. Nearly 50% underestimation can occur in high altitude regions with snow cover [50].

The observed trends in satellite-derived UVB are also impacted by deviations in absorbing aerosols, surface albedo, and cloud cover. While we observed small changes in UVR over time, deviations in these satellite input parameters may either exaggerate this trend or render a significant trend negligible even though one exists. Although documented limitations exist in satellite estimates, trends in these values have displayed correlation coefficients upwards of 98% when compared to ground-based UV meters [47]. Observed differences in satellite estimates should reflect non-differential misclassification in lifetime ambient sun exposure assessment. That is, all individuals in the study would have the same probability of exposure measurement error leading to an underestimation of UVB associations with health outcomes.

The OMI system was designed as an update to TOMS, but as previously discussed these satellites have differences in capturing input parameters within the erythemal UVB algorithm. Long-term trend analyses combining data estimated by these satellites must carefully consider the implications of joining these time series. We completed both long-term and separate stratified analyses by satellite type in order to distinguish the time periods of largest UV change and provide a comparison for the long-term analysis. Using either method, the estimated trends in this study were many orders of magnitude less than estimated lifetime ambient UVR in previous epidemiological studies [21,22,30].

In this study, we saw small trends in erythemal UVB from 1978 to 2014 which represents the extent of the climatological satellite record. As previously mentioned, accurate UVR exposure assessment for many UVR-associated outcomes will rely on a good assessment of childhood and young adult exposures. One assumption in our interpretation of these findings is that trends from 1978 to 2014 will hold pre-1978 for the lifetime of individuals living today with exposure histories that pre date the record. Other climatological analyses have shown this assumption to hold, with erythemal UVB increasing less than 5% relative to the 1975–1985 mean from 1960 to 2000 before decreasing slightly since then for the locations between 60° N and 30° N latitude [63]. If pre-1978 trends are 10%–15% larger than the 1978–2014 trends reported here, then the effect on lifetime ambient erythemal UVB would be minimal for the 80-year-old individual previously described living in Nevada (2.48–2.55 vs. 2.32).

Sun exposure assessment in humans has been addressed through a variety of methods in the epidemiological literature [4,76]. Some advanced methods use electronic dosimeters to measure personal UVR exposure. For large scale studies, this is impractical due to cost and length of follow-up time necessary in longitudinal analyses. Other objective methods include circulating vitamin D levels, auto-florescence, latitude of residence, approximations based on human geometry, and ambient UVR, but each of these have their biases [4]. Other subjective methods include sun diaries, location of residence and time spent outdoors in the sun, but these are subject to recall bias especially with exposures in the distant past [4]. Ambient erythemal UVB can be used as a proxy estimate, but use can be difficult due to data availability issues, perceived difficulty of use, size and complexity of data files, and computing time necessary. Often more crude proxies of UVR have been used. When considering the relationship between these crude proxies and UVR, latitude alone explains 15% of personal UVR variation [77], while a 1000-m increase in altitude corresponds to a 27% increase in erythemal UVB [78], cloud cover displays an inverse relationship with UVR [79]. Temperature may be a better proxy than others based on cloudiness as temperature influences the type of clothing worn. The relationship between temperature and UVR may be complex. Globally, annual mean erythemal UVB is highly correlated with annual mean temperature (*r* = 0.845), but locally significant correlations only exist in the Southern US states where annual temperature variation is low [80]. Future studies should assess the degree of exposure misclassification added by using these crude proxies for lifetime ambient erythemal UVB compared with the remotely sensed estimates shown here. Valid sun exposure assessment may rely on multiple methods including objective methods, subjective methods, and crude climatological proxies.

Investigators seeking to enhance UVR exposure assessment are increasingly adding more advanced techniques such as ambient erythemal UVR estimation [3,21,22,23,30,81,82,83], rather than sole use of subject recall or climatological proxies. In studies using erythemal UVB or similar objective UVR modeling techniques, recall bias due to UVR exposure estimation has been dramatically reduced, if not eliminated. Finding only small changes in UVB over the study period further supports the use of satellite data for outcomes with latency periods that might predate the satellite record. Although satellite UVR can be used to estimate ambient UVR exposure, satellite UVR does not provide information on exposure to an individuals’ skin. Models that combine ambient measures with aspects of individual level dosing (time spent outdoors, clothing worn, and skin sensitivity to the sun) will further reduce misclassification of exposure. Satellite UVR estimates should be added to more both more traditional models including human skin sensitivity, skin color, gender, age, personal protective behaviors, and relative time spent outdoors in the sunshine for UVR exposure assessment. These models can be further enhanced by more contemporary 3D models that account for human body cover and body surface available to receive estimated UVR [84]. As individuals born after the satellite record age, we will measure the effects of UVB exposures more effectively, but based on the results found here current approaches may still be reliable.

## 5. Conclusions

TOMS and OMI UVR estimates have been previously used to estimate sun exposure in various epidemiological studies including those focused on multiple sclerosis [16], cancer [23,24,25,26,27,28,29,30], asthma and hayfever [31], diabetes [32], and mortality [33,34]. Many of these studies assumed no long-term changes in UVR, and simply averaged UVR to estimate sun exposure. If satellite-derived UVR estimates have changed appreciably over time, then assumptions made from previous exposure studies may invalidate conclusions drawn from these analyses.

We investigated the temporal trends in satellite-derived erythemal UVB. Observed changes in erythemal UVB from 1978 to 2014 from satellites were not relevant for human exposure studies as they represented smaller changes than are necessary for appreciable misclassification of lifetime sun exposure based on current UVR exposure literature.

The trends seen could be impacted by factors unaccounted for in the analysis including ozone and climatological factors, as well as errors in the satellite UVR estimation. These results should be interpreted with caution as they rely on long-term estimates from satellite sensors with well documented intra-satellite and inter-satellite validation inconsistencies with ground level sensors. Satellite estimates will approximate ground level sensors in regions where clouds, absorbing aerosols, and snow cover are absent. The results of this analysis may be most appropriately generalized to these regions.
